# Metabolomic Footprint of Disrupted Energetics and Amino Acid Metabolism in Neurodegenerative Diseases: Perspectives for Early Diagnosis and Monitoring of Therapy

**DOI:** 10.3390/metabo13030369

**Published:** 2023-03-01

**Authors:** Patrycja Maszka, Magdalena Kwasniak-Butowska, Dominik Cysewski, Jaroslaw Slawek, Ryszard T. Smolenski, Marta Tomczyk

**Affiliations:** 1 Department of Biochemistry, Medical University of Gdansk, 80-210 Gdansk, Poland; 2 Division of Neurological and Psychiatric Nursing, Medical University of Gdansk, 80-211 Gdansk, Poland; 3 Department of Neurology, St. Adalbert Hospital, 80-462 Gdansk, Poland; 4 Clinical Research Centre, Medical University of Bialystok, 15-276 Bialystok, Poland

**Keywords:** biomarker, neurodegeneration, energy metabolism, Alzheimer’s disease, Parkinson’s disease, Huntington’s disease

## Abstract

The prevalence of neurodegenerative diseases (NDs) is increasing due to the aging population and improved longevity. They are characterized by a range of pathological hallmarks, including protein aggregation, mitochondrial dysfunction, and oxidative stress. The aim of this review is to summarize the alterations in brain energy and amino acid metabolism in Alzheimer’s disease (AD), Parkinson’s disease (PD), and Huntington’s disease (HD). Based on our findings, we proposed a group of selected metabolites related to disturbed energy or mitochondrial metabolism as potential indicators or predictors of disease. We also discussed the hidden challenges of metabolomics studies in NDs and proposed future directions in this field. We concluded that biochemical parameters of brain energy metabolism disruption (obtained with metabolomics) may have potential application as a diagnostic tool for the diagnosis, prediction, and monitoring of the effectiveness of therapies for NDs. However, more studies are needed to determine the sensitivity of the proposed candidates. We suggested that the most valuable biomarkers for NDs studies could be groups of metabolites combined with other neuroimaging or molecular techniques. To attain clinically applicable results, the integration of metabolomics with other “omic” techniques might be required.

## 1. Introduction

A group of congenital or acquired progressive diseases of the nervous system that result in the pathological loss of nerve cells is called neurodegenerative diseases (NDs). NDs follow a process of neurodegeneration leading to the signal transmission pathways damaging [1]. One of the common pathways of neurodegeneration is protein aggregation in the nerve cells. Depending on neurodegenerative disease, protein aggregates vary in composition and location in the nervous system. In Alzheimer’s disease, there are senile plaques mainly made of beta-amyloid or neurofibrillary tangles, mostly made up of hyperphosphorylated tau protein. In Parkinson’s disease, so-called Lewy bodies (LB) are formed, mainly containing primarily alpha-synuclein, while in Huntington’s disease, protein aggregates rich in polyglutamine residues are formed (called huntingtin) [2,3].

The number of patients with NDs is growing disproportionally to the previous expectations. For example, the number of patients with Parkinson’s disease will be doubled in the next 15 years to 12 million cases worldwide due to increased age, industrialization, and pollution [4]. Moreover, according to a report by the World Health Organization, Alzheimer’s disease nowadays affects almost 50 million people. By 2050, as predicted, 150 million people will be affected (with an estimated prevalence of 600 per 100,000 people worldwide) [5].

Although NDs have been known for many years, their causes, as well as diagnostics and prediction markers, have yet to be fully developed. Thus, this review will take a closer look at the pathophysiology at the molecular level of three selected neurodegenerative diseases: Alzheimer’s disease (AD), Parkinson’s disease (PD), and Huntington’s disease (HD), focusing mainly on disrupted brain energy metabolism, and finally present compounds related to them as new possible biomarkers or predictors of neurodegenerative diseases.

### Brain Energy Metabolism

**Figure 1:**
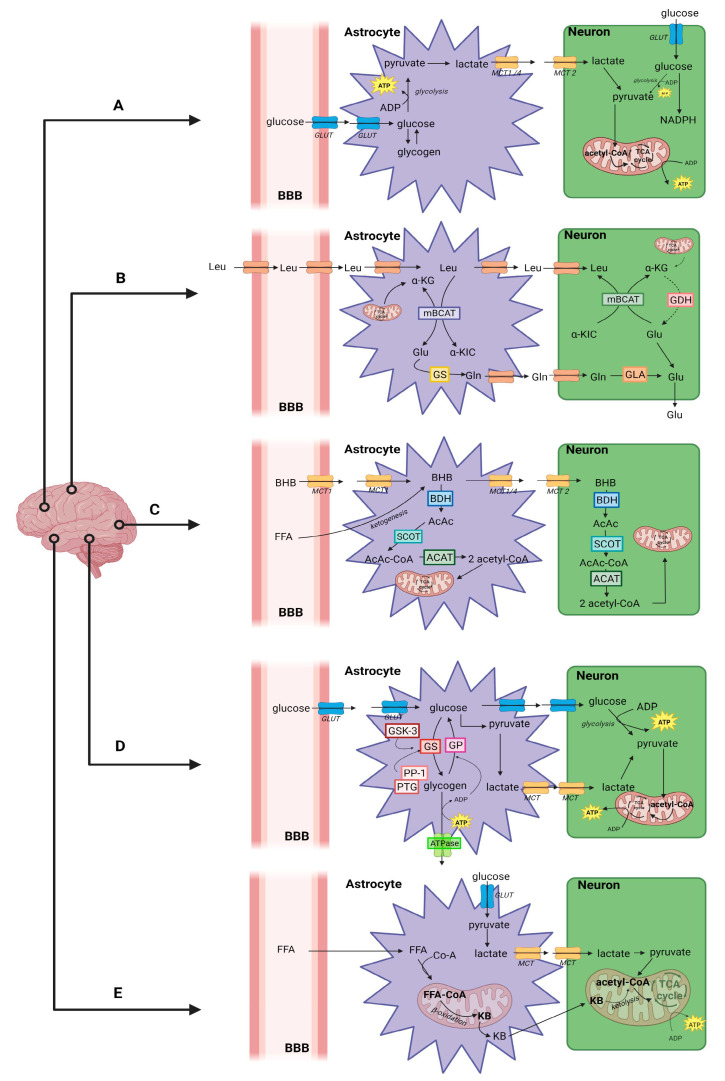
Brain energy metabolism: A—Glucose and lactate metabolism: Via the glucose transporter (GLUT), glucose crosses the blood–brain barrier (BBB) and is taken up by astrocytes. In the astrocyte, glucose can be stored as glycogen or metabolized by glycolysis. Pyruvate (a product of glucolysis) is converted by lactate dehydrogenase (LDH) to lactate, which is then transported by monocarboxylate transporters (MCT)1,2 and 4 from astrocytes to neurons. In the neurons, lactate is converted back by LDH into pyruvate, which is transferred to the mitochondria for aerobic energy production via the tricarboxylic acid (TCA) cycle. Glucose, which is directly taken up by neurons, can be used as an energy source in glycolysis or is transported to the pentose–phosphate pathway (PPP) for NADPH production [43]. B—Metabolism of branched-chain amino acids: Leucine (Leu) is delivered to astrocytes and neurons via transporters. In astrocytes, Leu is transaminated by mitochondrial branched-chain aminotransferase (mBCAT), resulting in glutamate (Glu) and α-ketoisocaproate (α-KIC). α-ketoglutarate (α-KG) is formed by de novo synthesis to replace the Glu carbon lost by the transformations in the TCA cycle. α-KIC is transported from astrocytes to neurons, Glu is converted to glutamine (Gln) by glutamine synthase (GS), while in the neuron Gln is converted back to Glu by glutamine synthase (GLA) for neurotransmission. In the neuron, the cycle of the branched-chain amino acid (in this case Leu) is completed by nitrogen transfer from Glu to α-KIC by cytosolic branched-chain aminotransferase (cBCAT). The resulting Leu is returned to the astrocytes. α-KG is aminated by glutamine dehydrogenase (GDH) to reform Glu. The cycle transfers nitrogen to neuronal Glu, and the cBCAT arm itself can buffer Glu in the neuron (preventing excitotoxicity accumulation) [44]. C—Metabolism of ketone bodies: Beta-hydroxybutyrate (BHB) is transported across the BBB by MCT transporters or is delivered to astrocytes in the fatty acid ketogenesis (FFA) pathway. BHB can then be transported to the neurons or be converted to acetoacetate (AcAc) by betahydroxybutyrate dehydrogenase (BHD). AcAc is catabolized to acetoacetyl-CoA (AcAc-Co-A) by succinyl-CoA3:-ketoacid coenzyme A (SCOT) transferase. AcAc-Co-A is then converted to 2-acetyl-CoA by the action of thiolase (ACAT). Exactly the same conversion takes place in neurons. Neurons are more efficient at oxidizing ketones than astrocytes [29]. D—Glycogen metabolism: Via GLUT, glucose from the BBB is transported into astrocytes. In astrocytes, glucose is converted into glycogen by glycogen synthase (GS), which is activated by GS kinase-3 (GSK-3). Glycogenolysis is mediated by glycogen phosphorylase (GP). GS is activated by protein phosphatase-1 (PP-1), and both GS and PP-1 interact with glycogen via protein targeting to glycogen (PTG). Increased adenosine triphosphate (ATP) consumption by the sodium-potassium (Na, K) ATPase required for glutamate uptake activates glycolytic enzymes. Glycolysis in astrocytes leads to the production of lactate, which serves as an energy substrate for oxidative metabolism in active neurons [45]. E—Fatty acid metabolism: Fatty acids (FFAs) are delivered to astrocytes by crossing the blood–brain barrier. Once inside astrocytes, FFAs are transported to the mitochondrial matrix, where they undergo β-oxidation, resulting in the production of ketone bodies (KBs). The KBs are then transported to neurons, where they undergo ketolysis in the mitochondrial matrix, producing energy [40].

The adult brain has high energy requirements. However, the brain represents only 2% of the total human body mass, and about 20% of the oxygen and 25% of the glucose consumed by the human body are dedicated to preserving cerebral functions. The main processes leading to that high energy demand are maintaining the ion gradient homeostasis (dissipated by signaling processes such as postsynaptic and action potentials) and uptake and recycling neurotransmitters [6,7]. It is well known that there is a close relationship between brain activity, glutamatergic neurotransmission, energy requirements, and glucose utilization. However, glucose is the primary and critical energy substrate for the brain. Brain cells can efficiently utilize various energy substrates, including lactate, pyruvate, glutamate, glutamine, branch-chain amino acids, and ketone bodies (Figure 1). Most of these metabolic substrates are formed using glucose as the carbon source. Because brain metabolism is a compartmentalized process, there is a constant intercellular movement of these substances within the brain cells [8,9]. It is important to note that both astrocytes and neurons can fully break down glucose and/or lactate. Additionally, both types of cells have similar numbers of mitochondria, and therefore similar metabolic potential. However, neurons and astrocytes tend to use different metabolic pathways under physiological conditions, partly due to cell-specific patterns of gene expression related to energy metabolism [8,10].

Following their high energy requirements, neurons have a higher rate of oxidative metabolism than glial cells [11,12]. Interestingly, a great deal of evidence shows that neurons can efficiently use lactate as an energy source [13] and even show a preference for lactate over glucose when both are available [14]. Some studies have provided insights into the mechanisms behind these neuronal features. It has been reported that the enzyme 6-phosphoglucose-2-kinase/fructose-2,6-bisphosphatase-3 (Pfkfb3) is virtually absent in neurons due to its constant proteasomal degradation, which is in contrast to the high expression levels observed in astrocytes [8,9]. This enzyme is responsible for the production of fructose-2,6-bisphosphate (fructose-2,6-P2), a potent activator of the glycolytic enzyme phosphofructokinase-1 (PFK). As a result of the low production of fructose-2,6-P2, neurons have a slower glycolytic rate and, unlike astrocytes, are unable to increase this pathway in response to nitric oxide-induced cellular stress [15,16]. The activation of neuronal glycolysis through Pfkfb3 overexpression leads to oxidative stress and apoptosis [15], suggesting that neurons are unable to sustain a high glycolytic rate. On the other hand, astrocytes have a different specific gene expression profile that leads to a high glycolytic rate and preference for the production and release of lactate over the entry of pyruvate in the tricarboxylic acid (TCA) cycle [17,18]. This is achieved through the regulation of Pfkfb3, aspartate/glutamate carrier (AGC), and pyruvate dehydrogenase, which work in combination to maintain a high nicotinamide adenine dinucleotide (NAD)+/NADH ratio and ensure the maintenance of a high glycolytic rate [19,20,21]. These metabolic characteristics of astrocytes play a crucial role in maintaining energy homeostasis in the brain, providing neurons with lactate as an energy substrate and participating in recycling neurotransmitters through the glutamate–glutamine cycle (which is described in detail below).

As a result, neurons and astrocytes have distinct but complementary metabolic profiles which allow for unique metabolic cooperation, which is called the astrocyte–neuron lactate shuttle. The essence of this model is that: (1) neuronal activity increases extracellular glutamate (via glutamatergic neurotransmission), which is avidly taken up via a Na+-dependent mechanism by specific glial glutamate transporters; (2) the resulting increase in [Na+] and activates the Na+/K+ ATPase (in particular by mobilizing its alpha2 subunit), thereby increasing adenosine triphosphate (ATP) consumption [19], glucose uptake, and glycolysis in astrocytes; (3) this, in turn, leads to a significant increase in the production of lactate which is released in the extracellular space; and in the last step (4): lactate can be used as an energy substrate for neurons for oxidative-derived ATP production [20,21]. Neurons can also take up glucose through the neuronal glucose transporter 3 (GLUT3). Additionally, astrocytes participate in the recycling of synaptic glutamate through the glutamate–glutamine cycle. After being taken up by astrocytes, glutamate is converted to glutamine by the action of glutamine synthetase and transported to neurons, where it is converted back to glutamate by glutaminases [8].

Another amino acid group that seems important to maintain proper brain function is branch-chained amino acids (BCAAs). The BCAAs such as leucine, isoleucine, and valine are important for various critical biochemical processes in the brain, including protein synthesis, energy production, the compartmentalization of glutamate, synthesis of the amine neurotransmitters from aromatic amino acids—serotonin from tryptophan and the catecholamines—dopamine and norepinephrine, which are derived from phenylalanine, and tyrosine [22]. BCAAs are transported into the brain by a transporter, located at the blood–brain barrier (BBB) on central nervous system capillary endothelial cells, which is almost entirely saturated at normal plasma amino acid concentrations, competitive, and shared by several large neutral amino acids (LNAAs), including the aromatic amino acids, tryptophan and phenylalanine [23,24]. Due to these relations, their increased uptake into the brain resulted in decreased brain uptakes and levels of aromatic amino acids [25]. Thus, BCAA can modulate levels of neurotransmitters produced in the brain, altering brain function and behavior [24].

In the event of glucose deprivation, such as during prolonged fasting, intense exercise, or pathological conditions such as diabetes, the brain can utilize ketone bodies as an alternative source of energy [26]. The transport of ketone bodies across the BBB is mediated by specific carrier proteins, known as monocarboxylate transporters (MCTs). Unlike glucose transport, which is dependent on neuronal activity, the uptake of ketone bodies is primarily determined by their concentration in circulation [26]. MCTs are the only known transporters for ketone bodies and are distributed throughout the brain [27]. Studies in rodent models have shown that the expression of MCTs is distinct between cell types, with the MCT1 isoform located at the blood–brain barrier on endothelial cells and astrocytes, and MCT4 expressed in astrocytes as well [28]. In contrast, neurons predominantly express the MCT2 isoform, which has a high affinity for beta-hydroxybutyrate (BHB). The expression of MCT2 in neurons is localized to mitochondria-rich postsynaptic density structures, suggesting that the transporter plays a significant role in synaptic transmission [27,29]. Ketone bodies (beta-hydroxybutyrate (BHB) and acetoacetate), once transported into the brain, are converted back into acetyl-CoA, which enters the tricarboxylic acid (TCA) cycle to generate ATP. This conversion occurs in the mitochondria, where BHB is transformed into acetoacetate by the enzyme BDH, producing NADH. Acetoacetate is then converted into acetoacetyl-CoA by the enzyme succinyl-CoA:3-ketoacid coenzyme A transferase (SCOT) and then back into two acetyl-CoAs by thiolase, which enters the TCA cycle. Unlike glucose oxidation, this conversion does not require ATP [30]. Studies in developing rodent brains have shown that neurons, astrocytes, and oligodendrocytes are all able to use ketone bodies for oxidative metabolism more efficiently than glucose. However, neurons and oligodendrocytes seemed to be more efficient in oxidizing ketones than astrocytes [31].

Brain cells, primarily astrocytes, can store glucose in the form of glycogen [32]. The regulation of glycogen levels in astrocytes follows the same pattern as seen in the liver and muscle, through glycogenesis and glycogenolysis. Its breakdown results in glucose-6-phosphate, which can enter glycolysis and generate pyruvate, leading to the production of ATP through aerobic oxidation [33]. Brain glycogen concentration is relatively low compared to the liver and muscle [34]. Glycogenolysis not only helps to commonplace the energy supply but also satisfies special requirements such as higher local energy demand induced by regional stimulation, stability maintenance during hypoglycemia, responding to rapid and high-demand needs signaled by neuromodulator factors such as norepinephrine, drug addiction, memory formation, and consolidation, as well as sleep and development [35]. Thus, its dysfunction has been implicated in memory impairment, depression, and epilepsy [34]. Moreover, it is known that glycogen can act as both a regulator of glycosylation and a substrate for glycophagy. The degradation of brain glycogen into glucosamine-6-phosphate leads to the formation of uridine diphosphate N-acetylglucosamine, a substrate for O-linked N-acetylglucosaminyltransferase to produce glycosylated proteins [36]. On the other hand, glycogen can also be degraded through glycophagy, a form of chaperone-mediated autophagy in lysosomes. It moves into the phagophore under the guidance of starch-binding domain-containing protein 1 (STBD1) and γ-aminobutyric acid receptor-associated protein-like 1 (GABARAPL1), and thereafter it is delivered to lysosomes where it is broken down by acid α-glucosidase into glucose [37]. Dysfunctional glycophagy-related glycogen accumulation is the cause of neurodegeneration in Lafora disease. It indicates that glycophagy is necessary for maintaining cellular survival in the brain [38].

Under conditions of fasting, prolonged starvation, or diabetes, the brain can utilize fatty acid (FA) to meet its energetic demands. Recent research has indicated that FA oxidation accounts for approximately 20% of the total energy consumption of the human brain [39]. The primary source of FFAs that cross the BBB is believed to derive from nonesterified long-chain FFA/albumin complexes, which dissociate from albumin and circulating lipoproteins. Once they have entered cells, acyl-CoA synthetases convert them to acyl-CoA, allowing for their intracellular entrapment. After entering astrocytes, FFAs are translocated into the mitochondrial matrix for β-oxidation and ketone bodies production. Ketone bodies are converted into acetyl-CoA, which in turn enters the TCA for energy production. It is worth noting that glucose is necessary to provide substrates for the Krebs cycle (such as succinyl-CoA) for complete ketone bodies oxidation. Moreover, peroxisomal α- and β-oxidation can also metabolize branched- and very long-chain FFAs [40]. FFAs can also trigger a range of detrimental activities within the cell. Active neuronal cells produce excessive FFAs, which they are unable to use to promote oxidative ATP synthesis, resulting in the accumulation of toxic FFAs in neurons. These FFAs must be stored in intracellular lipid droplets (LDs) as triglycerides to prevent neuronal damage. Excess FFAs are transported by apolipoproteins into astrocytes, which are rich in LDs and less vulnerable to harmful reactive oxygen species (ROS) activity than neurons [39]. Astrocytes are believed to be the primary sites of FFAs storage and metabolism in the brain. LDs act as energy storage centers, transporting FFAs to mitochondria during nutrient depletion and serving as an alternative energy source. Therefore, to protect neurons from FFA-related lipotoxicity and meet the energy demand in specific situations, FFA storage and oxidation processes appear to rely on a tight metabolic interplay between neurons and astrocytes [41].

Besides typical energy metabolic substrates, purines also play a crucial role in the cellular metabolism of the central nervous system, acting as signals to control cell growth and provide energy to the cell. The balance of nucleotides is maintained by a continuous supply of preformed purine and pyrimidine rings, mainly in the form of nucleosides, that can enter the brain through the blood–brain barrier or can be locally supplied by the conversion of extracellular nucleotides (by extracellular nucleotidases located in the neuronal plasma membrane) [42]. Thus, changes not only in energy metabolism but also in purine metabolism and signaling (that also could be caused by impaired energy metabolism) may impact neuronal function.

It has to be mentioned that the neurons susceptible to degeneration need more energy to maintain their structural and functional integrity. Therefore, any disruption in cellular energy metabolism leads to enormous energy demand that eventually results in oxidative stress that (with an impaired intracellular protein aggregates removal) may be deleterious to the development of neurodegeneration.

## 2. Alzheimer’s Disease

Alzheimer’s disease (AD) is a chronic condition characterized by cognitive decline and neuropsychiatric symptoms that leads to behavioral disorder and dependency [46,47,48]. The average life expectancy after a patient is diagnosed with AD is about seven years, while only 2.4% of patients survive more than 14 years [49,50].

AD symptoms are caused by the loss of nerve cells and their connections in the cerebral cortex and some subcortical regions. As a result of AD the decreased number of neurons is noted in the temporal lobes, parietal lobes, and parts of the frontal lobes, as well as the cingulate gyrus. Senile plaques in AD are formed by the aggregation of small beta-amyloid peptides (Aβ) numbering between 39 and 43 amino acid residues, a fragment of the amyloid precursor protein (APP) formed by the action of the proteolytic enzymes beta and gamma-secretase on APP. APP is a transmembrane protein that penetrates the cell membrane surrounding the axon. This protein is critical in neuronal cell growth, survival, and damage repair [51,52,53,54].

Another abnormal mechanism that leads to AD progression is tauopathy, associated with tau protein aggregation, found almost exclusively in nerve cells. From a biochemical point of view, tau protein is a desialylated (lacking sialic acid) transferrin. Phosphorylated tau protein is bound to microtubules to form a microtubule-associated protein (MAP), which is one of the building blocks of the neuronal cytoskeleton, responsible for its stabilization and binding to neurofilaments and cell organelles. Microtubules in the cell are involved in the transport of substrates and metabolites of the cell’s energy metabolism from the perikaryon to the axons and back [55]. The protein’s affinity for microtubules depends on its degree of phosphorylation, while hyperphosphorylation results in neurofibrillary tangles (NFT) visible in the cytoplasm of neurons [55,56]. These degenerations appear in the brainstem, while with the progression of the disease they are noticeable in the hippocampus and eventually in the sensorimotor areas [57]. The mechanisms of both pathologies may arise independently of each other. However, evidence suggests that Aβ aggregation causes tauopathy progression, not the other way around. Furthermore, the disturbance of the BBB and cerebral amyloid angiopathy in AD was noted [58].

It has to be mentioned that one of the major risk factors for AD is aging. Interestingly, some similarities in glucose metabolism-related proteins have been observed in the brain during normal aging and AD [59]. Moreover, the presence of one of the apolipoprotein E (ApoE) alleles, ApoE4, is linked with a higher risk of developing late-onset AD [60]. On the contrary, the Apo-E2 allele decreases AD risk [61]. Specific rare mutations in presenilin-1 (*PSEN-1*), presenilin-2 (*PSEN-2*), and Amyloid Precursor Protein (*APP*) genes are responsible for the autosomal dominant disease variant, characterized by an early onset of symptoms [62]. Modifiable risk factors are low education level, physical inactivity, brain injuries, smoking, alcohol abuse, depression, and social isolation [63]. Cardiometabolic factors, such as hypertension, diabetes, obesity, or hypercholesterolemia, are also involved in AD development [62,63]. Those data suggested that disrupted metabolic pathways may contribute to AD pathophysiology and progression. It is interesting to note that studies have shown that microwaves may cause adverse effects such as memory loss and decreased learning ability, which are observed in patients with Alzheimer’s disease. With the increasing number of radiant devices, microwaves may also be considered a potential risk factor of AD [64]. 

The diagnostic process should start with obtaining a detailed medical history from a patient and caregivers. Following that, potentially reversible causes of dementia, i.e., hypothyroidism, vitamin B_12_ deficiency, or depression, must be excluded. Physical and neurological examinations and psychological assessments should be conducted. For magnetic resonance imaging (MRI) scans, typically scans for AD medial temporal lobe atrophy can be seen, and other structural brain pathologies leading to cognitive impairment can be excluded [65]. Posterior cingulate and temporoparietal hypometabolism on fluorodeoxyglucose-PET (FDG-PET) and cortical Aβ depositions in Amyloid Pittsburgh compound B-PET (PiB-PET) are supportive neuroimaging markers. Although tau depositions visible in tau-PET scans allow us to differentiate between AD phenotypes and other tauopathies, this method is not commonly used in clinical practice [62]. The assessment of proteins correlated with AD in cerebrospinal fluid (CSF) is another diagnostic tool. CSF levels of Aβ 42 in AD patients are decreased, whereas phosphorylated-tau and total-tau levels are elevated [66]. Only symptomatic treatment is available. To treat mild to severe dementia, acetylcholinesterase inhibitors (donepezil, rivastigmine, galantamine) and N-methyl D-aspartate receptor antagonists (memantine) are used. Non-pharmacological treatment, such as a healthy diet, physical activity, and cognitive training, may also be helpful [67].

### 2.1. Disrupted Energy Metabolism in AD-Affected Brain

Energy hypometabolism and, in particular, a decrease in glucose metabolism is one of the first dysfunctions observed in AD patients [22,68]. These changes can affect learning and memory abilities by causing problems with neurotransmission maintenance. Specific abnormalities of glucose metabolism occur in the posterior cingulate and temporoparietal cortex [69]. In addition, there is a significant decrease in glucose uptake at the site of early Aβ deposition, even 10 years before the onset of the first symptoms [70]. Moreover, regional hypometabolism in the brain is a predictor for progressive cognitive decline, and reduced cerebral metabolism is associated with carriers of the AD risk allele of the APOE-4 gene [61,71]. Aβ also interacts with insulin receptors (IR) and glucose transporter (GLUT), resulting in impaired insulin signaling. AD patients show a decrease in GLUT1 and GLUT3 proteins, as their amount is closely correlated with the amount of glucose consumed. This leads to the inhibition of the insulin (IIS) and IGF-1 signaling pathways, ultimately damaging mitochondrial function and structure [72].

Changes in the structure and function were noted in patients. It is believed that damaged mitochondria may contribute to, or even drive, the progression of AD [73]. Mitochondria isolated from AD patients display reduced enzymatic activity of cytochrome C oxidase, which is part of the respiratory chain complex IV [74]. Interestingly, both Aβ and AβPP have been observed to colocalize with mitochondria through binding to alcohol dehydrogenase [73]. The precursor protein Aβ partially passes through the mitochondrial protein transport apparatus while blocking it. As a result of this blockage, cytochrome oxidase (COX) activity decreases [75]. Furthermore, it is described that progressive accumulation of Aβ in mitochondria is associated with reduced oxidative respiration and reduced activity of the rate-limiting TCA cycle enzyme, α-ketoglutarate dehydrogenase complex, and the pyruvate dehydrogenase complex (PDHC), which generates acetyl-CoA for entry into the TCA cycle [76]. These findings suggest that metabolic dysfunction and mitochondrial Aβ accumulation may occur early in AD progression, preceding the formation of extracellular plaques.

The occurrence of senile plaques disrupts the homeostasis of calcium metabolism. It is assumed that Aβ can form pores in the plasma membrane, permeable to calcium ions [77]. Pore formation is promoted by phosphatidylserine on the cell surface, which can be an indicator of cells that are undergoing apoptosis [78]. A pore-forming mechanism is involved in the neurotoxicity of senile plaques, as calcium handling disruption results in increased free radical formation in the cytosol, [79] causing damage to the mitochondria and a decrease in ATP levels. In addition, there is an inhibition of the oxidative chain which depolarizes the mitochondria, further impairing the calcium metabolism of the cell [77]. Impaired mitochondrial function decreases adenosine monophosphate-activated protein kinase (AMPK) activity, which is another factor in the progression of bioenergetic abnormalities in the brain of AD patients [80]. Long-term dysregulation of calcium ion influx, resulting in its unrestricted entry into mitochondria, causes excessive accumulation of reactive oxygen species (ROS), whose increased levels induce the transcription of pro-inflammatory genes and the release of cytokines (e.g., interleukin-1β (IL-1β), IL-6, and tumor necrosis factor alpha (TNFα)), as well as chemokines that cause neuroinflammation [77].

The other impaired metabolic pathway in AD is the kynurenine pathway (KP). It is responsible for the metabolic breakdown of tryptophan, resulting in various biologically active intermediate metabolites. One branch of this pathway leads to the synthesis of NAD+, an indispensable cofactor that is involved in numerous enzymatic redox reactions and plays a crucial role in mitochondrial energy generation [81]. Indeed, in the TCA cycle and glycolysis, many individual reactions are highly regulated by the availability of NAD+. The activity of enzymes such as glyceraldehyde-3-phosphate dehydrogenase (G3PDH), lactate dehydrogenase (LDH), and the pyruvate dehydrogenase (PDH) complex in glycolysis requires NAD+. Similarly, in the TCA cycle, NAD+ is required by enzymes such as malate dehydrogenase (MDH), α-ketoglutarate dehydrogenase (α-KGDH), and isocitrate dehydrogenase (IDH), and also plays a role in regulating complex I [82]. In the brain of AD patients, the rate-limiting enzyme in KP, indoleamine 2,3-dioxygenase (IDO), induces the formation of two neurotoxic metabolites as end products of this pathway. These metabolites are quinolinic acid (QUIN) and 3-hydroxyquinurenine (3-HK). Kinurein in microglia cells is transformed to 3-HK, which induces oxidative damage, allowing the entry of QUIN, which is excitotoxic and neurotoxic [83,84]. Inflammation and high levels of inflammatory cytokinins cause QUIN to be produced in excess, and instead of being converted to NAD+ to protect neurons, it becomes saturated, leading to neuronal apoptosis [85].

### 2.2. Selected Candidates for Biomarkers Related to Disrupted Energy Metabolism Pathways and Intracellular Homeostasis Imbalance

**Figure 2:**
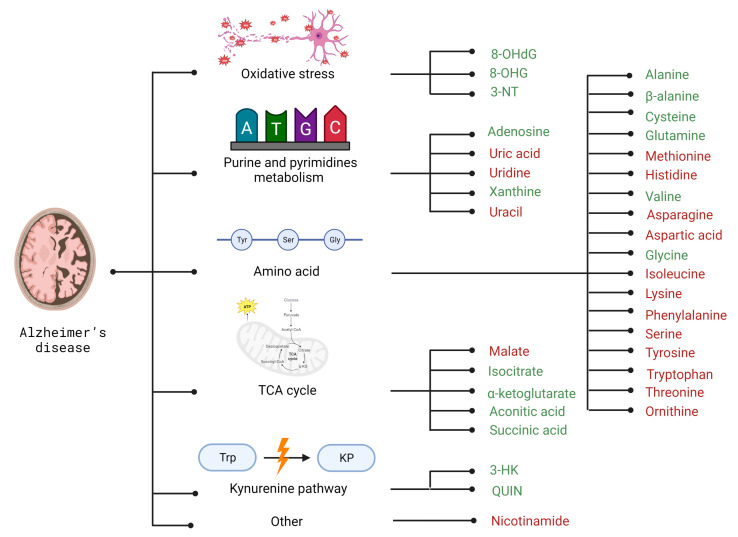
Biomarkers of PD related to energy metabolism disruption (green—increased levels, red—decreased levels).

As described above, changes in the brain’s energy metabolism may result in deterioration in the peripheral concentration of its related metabolites. Through metabolomic studies, changes in amino acids (alanine, β-alanine, cysteine, L-glutamine, methionine, histidine, valine, asparagine, aspartic acid, glycine, isoleucine, lysine, phenylalanine, serine, tyrosine, tryptophan, threonine, ornithine, uracil), as well as TCA cycle metabolites (malate, isocitrate, and α-ketoglutarate), oxidative stress indicators (3-Nitrotyrosine, 8-hydroxyguanosine), and other metabolites (linked with purine and pyrimidines metabolism like adenosine, uric acid, xanthine and uridine, or kynurenine pathway like 3-hydroxyquinurenine) were highlighted in Alzheimer’s disease patients [86,87,88,89] (Figure 2).

Alanine is synthesized from pyruvate in a reaction catalyzed by alanine aminotransferase (ALT); this amino acid is a biosynthetic derivative of the intermediate of glycolysis, so its concentration can be correlated with pyruvate kinase activity [90]. Blood and cerebrospinal fluid (CSF) studies of AD patients show increased alanine compared to healthy subjects. Thus, it can be assumed that kinase activity decreases, which translates into increased NFT and amyloid pathology [87,90]. AD patients also show increased β-alanine levels in both CSF and plasma [87]. However, studies of serum from a cohort of elderly Japanese demonstrate that the risk of developing AD decreases significantly with increasing β-alanine levels. It is also hypothesized that β-alanine stories are strongly related to the amount of its metabolite, carnosine [91]. In this study, with a randomized group of Japanese and Polish people, supplementation with carnosine showed a beneficial effect on cognitive function. Thus, it is suggested that a decrease in serum β-alanine concentrations contributes to AD development. Therefore, this amino acid has a strong implication for becoming a biomarker of AD progression [91].

Besides alanine, another amino acid concentration might be helpful in AD diagnosis—cysteine. Cysteine is involved in the S-nitrosylation of proteins, and abnormalities in this process can lead to abnormal protein folding, mitochondrial degradation, and damage to synapses, ultimately leading to neuronal cell apoptosis [92]. Thus, an increase in the concentration of this amino acid in both CSF and plasma indicates a disruption of the S-nitrosylation process, and the resulting symptoms are closely correlated with those typical of AD [87]. An increase in cysteine may be the first signal to focus on diagnosing AD in a patient before the onset of symptoms characteristic of an advanced stage of the disease [92].

Another amino acid that distinguishes AD patients is the elevated urinary L-glutamine level [93]. Increased urinary levels of this amino acid may suggest disrupting the glutamine cycle, resulting in the excretion of glutamine from the body, preventing its conversion to glutamate. Glutamate deficiency results in impaired synaptic plasticity and impaired learning. Thus, its urinary levels may suggest disease progression [94]. In addition to L-glutamine, N-acryloyl glycine and isobutyl-L-carnitine, which are fatty acid metabolites, are also detected in the urine of AD patients. Recent findings show that fatty acid metabolism is also linked to AD progression [94].

Histidine is the next selected amino acid that exhibited significant differences in concentrations in AD patients compared to healthy controls. It has been observed to be decreased in the serum of AD patients [87]. It was also confirmed in an integrative metabolomics-genomic study (that analyzes genetic, transcriptomic, metabolomic, and proteomic data) in AD [95]. Neuronal histamine (a monoamine synthesized from histidine) is key in maintaining alertness and controlling the diurnal rhythm [96]. A common symptom in AD patients is a disturbance in sleep and circadian rhythm [78]. During the day in healthy individuals, histidine levels are lower and higher at night, while the opposite is seen in AD patients, which would explain the restless sleep and apathy during the day. Regular histidine monitoring could allow for the detection of potential disease risks before developing typical AD symptoms [97].

Moreover, methionine, the amino acid that plays a key role in oxidative stress and neurotoxicity associated with β-synuclein aggregates, is observed to be decreased in the serum of AD patients [87]. Decreased concentrations of this amino acid might be caused by the oxidation of this amino acid to sulfoxide by Aβ aggregates. This reaction is irreversible and disrupts the function of methionine as a promoter of the secondary structure of the alpha helix, which promotes the formation of senile plaques. However, this process does not accelerate the progression of the disease but might be one of the first dysfunctions associated with it [98].

Other interesting metabolites found in AD are BCAAs. A study by Tynkkynen et al. analyzed data from 11 groups of 22,623 individuals with over 2000 cases of AD. The results showed that each of the three BCAAs (valine, leucine, and isoleucine) was linked to a reduced risk of dementia and AD [99]. These findings were supported by another study by Toledo et al., who reported slower cognitive decline and less brain atrophy in the Alzheimer’s Disease Neuroimaging Initiative (ADNI) cohort with higher levels of valine and suggested that changes in BCAA degradation may be linked to AD [86].

In AD, the TCA cycle is also disrupted, so direct and indirect metabolites of this pathway may also become potential biomarkers. An example of such a compound is isocitrate, whose serum levels in patients are elevated, as is the related α-ketoglutarate. These compounds indicate altered energy metabolism in AD patients. However, more studies are needed to highlight its significance as an indication of TCA impairment [87,100]. Additionally, Kalecký et al. also observed elevated levels of aconitic and succinic acids [101]. Another example of such a compound is acylcarnitines, whose plasma levels in AD patients are significantly higher than in controls. These compounds indicate disturbances in the transport of fatty acid or its oxidation. Some studies demonstrated lower plasma of AD patients, concentrations of glucogenic and ketogenic amino acids that indirectly enter the TCA cycle through the production of intermediate [102,103].

Due to the numerous mitochondrial damages and oxidative stress induced by the presence of senile plaques, already-defined biomarkers of oxidative stress could become potential markers of AD [103]. Patients with AD show features of oxidative stress early in the course of the disease, which further promotes these compounds to become markers of AD progression. Peroxidation and oxidation of biomolecules induced by oxidative stress generate stable compounds that can be detected in serum, plasma, and CSF. Interestingly, a biomarker of lipid peroxidation F2-isoprostane was also heightened in AD patients compared to healthy individuals of the same age as the patients [104]. 3-Nitrotyrosine (3-NT) is a stable compound determined from cerebrospinal fluid, and AD patients also show increased levels. As a result of oxidative stress, the tyrosine regions of the tau protein are nitrated [105]. The most common markers used to determine levels of oxidative stress are 8-hydroxy-2’-deoxyguanosine (8-OHdG) and 8-hydroxyguanosine (8-OHG), which are products of oxidation of DNA and RNA bases by hydroxyl radicals. An inversely proportional concentration of 8-OHG to the concentration of Aβ was observed. In addition, the levels of these compounds were five times higher than those of healthy subjects [106].

Disruption of the kynurenine pathway results in the formation of neurotoxic metabolites that pass through the BBB [107]. Levels of the kynurenine pathway substrate itself, tryptophan, were also determined and significantly reduced, while its degradation products, such as 3-HK and quinolinic acid, were significantly heightened. Both of these tryptophan metabolites are associated with central nervous system (CNS) neuroinflammation in AD. These catabolites are also strongly associated with disease severity and increased pathology in the patient’s brain, such as increased Aβ or increased phosphorylation of tau protein [102]. Interestingly, recently Dalmasso et al. highlighted significantly reduced levels of nicotinamide, one of the NAD+ metabolites, in comparison to cognitively regular participants. In addition, high plasma levels of nicotinamide showed a 27% risk reduction of progressing to AD dementia within the following 2.5 years; this hazard ratio is lost afterward [108]. It suggested that not only the kynurenine pathway but also the investigation of NAD+ and nicotinamide metabolites might be interesting in investigating new AD biomarkers.

As described above, purines and pyrimidines metabolism might also be linked with impaired energy metabolism and, thus, the development of neurodegeneration. Interestingly, adenosine, one of the adenine nucleotides catabolites, is an important metabolite that contributes to the metabolomic differentiation of AD patients from healthy individuals. High levels of adenosine in the brain are associated with the normal aging process; however, an additional increase is observed in the serum of AD patients [100]. Adenosine is a neurotransmitter that acts as a neuromodulator and neuroprotector by controlling glutamate release [109]. In neurons where β-amyloid is deposited or tau proteins are hyperphosphorylated, there is a redistribution and defective regulation of adenosine receptors in the frontal cortex of AD patients [87,100]. 

Another interesting example of a purine metabolite with significantly abnormal levels in AD is xanthine [110]. Molecular studies show that this compound may mediate protective effects, but more detailed studies are needed to prove its direct link to AD pathologies [87,111]. Interestingly, the last metabolite of adenine nucleotides catabolism, uric acid, seems to be interested in AD diagnosis. Uric acid is one of the most important oxidants in plasma. It is formed by the breakdown of not only ATP but also DNA or RNA [112]. Uric acid is responsible for regulating inflammasome activity, interleukin-1 beta release, or immune functions, including recognition of damaged cells [113]. Patients with AD have significantly lower serum uric acid levels, but it is unclear whether low uric acid levels are a consequence of AD or contribute to its progression. On the other hand, this metabolic feature is present in most AD patients, so detection of its cause and level in different stages of AD might be interesting [87,112].

Another metabolite whose change in concentration predisposes it to become a biomarker of AD is uridine. Uridine is a nucleoside and one of the precursors of phosphatidylcholine, which is the main component of the neuronal cell membrane [114]. Furthermore, it has been observed in cohort studies that lower uridine levels are associated with disease progression [87]. The decrease in uridine in patients may be explained by the fact that there is an increased demand for uridine due to the ongoing need to regenerate synaptic membranes damaged by AD pathologies [100,114].

It is accepted that AD-related pathological and molecular changes occur over a long period before the first symptoms are observed, providing ample opportunity to detect biological alterations in various biological samples that can aid in early diagnosis and modify treatment outcomes. In 2022, comparative metabolomics analysis integrating multiple data sources revealed the metabolomic similarities and differences between AD patients and animal models, confirming that the existing AD animal models have good commonality with AD patients at the metabolite level. Data obtained from AD experimental models may also be valuable in AD biomarkers studies. Interestingly, in this work, the role of disrupted levels of amino acids and their analogs, such as glycine, alanine, aspartate, glutamate, glutamine, serine, valine, tyrosine, and purine metabolite–hypoxanthine, were highlighted [115]. Nevertheless, it has to be mentioned that differences in amino acid profiles may be related to different dietary habits and, more specifically, protein intake between the compared groups. Thus, metabolites from this group might not be suitable for creating a diagnostic marker of NDs. Interestingly, in the study mentioned above, Dai Z et. al., using a metabolite-target network, predicted potential protein markers in AD [115]. One may conclude that reverse metabolomics marker typing studies based on proteomic and transcriptomic data may bring new clues and directions for research on metabolites in AD, including metabolites related to impaired energy metabolism.

## 3. Parkinson’s Disease

Parkinson’s disease (PD) is a chronic neurodegenerative disorder with cardinal motor symptoms (bradykinesia, rigidity, and rest tremor), along with a long list of non-motor problems (autonomic, neuropsychiatric, sensory) [116,117]. It is the second most common neurodegenerative disorder after AD, with a mean 0.3–0.6% prevalence worldwide and increasing with age (1.5% in the general population of people > 65) [118].

The main pathology of PD is related to neuronal death in the basal ganglia in the brain, which affects up to 70% of dopamine-secreting nerve cells in the pars compacta of the substantia nigra (SN) [119]. Along with the loss of dopaminergic nerve cells in the SN, there is a reduction in the number of astrocytes and an increase in microglia. Significant neuronal loss can also be observed in the locus coreleus, the basal nucleus of Meynert, the dorsal motor nucleus of the vagus nerve, as well as the hypothalamus and the olfactory bulb [120].

Neuronal damage is induced by cytotoxic aggregates of misfolded alpha-synuclein proteins (ASN), called Lewy bodies (LB) [2,121]. As a result of ASN interaction with negatively charged lipids, e.g., phospholipids from cell membranes, they induce the conformation of the protein in an α-helical structure. Thereafter, as a result of the numerous transformations with the disease progression, the protein changes to a β-harmonic structure, which promotes the formation of its aggregates. Transformation of the conformation results from phosphorylation of serine 129, shortening of the C-terminus, or ubiquitination. Depending on the pathology of the mechanism, different types of ASN can be observed in the brain—unfolded monomers, soluble oligomers, or insoluble fibrils. The most toxic form of ASN is oligomeric ASN, which increases and promotes abnormal protein folding [122,123,124,125].

Interestingly, the binding of ASN to major histocompatibility complex (MHC) receptors in inflammasomes results in the release of pro-inflammatory cytokines such as IL-6 IL-1β, IFNγ, and TNFα [126]. Activated microglia convert the protective phenotype of astrocytes to a neurotoxic one. In healthy brains, astrocytes protect neuronal connections, whereas in PD patients they lose the protective capacity of dopaminergic connections in the striatum. Microglia through MHC-I and MHC-II present antigens to CD4+ T lymphocytes, which, when activated, can cross the BBB, enhancing the release of pro-inflammatory cytokines. This process contributes to the breakdown of the BBB in PD patients [127].

PD pathogenesis is also accompanied by mitochondrial dysfunction. Experimental data indicating the presence of an impaired mitochondrial ubiquitin–proteasome system, which is responsible for the selective degradation of damaged or misfolded proteins, contribute to the pathogenesis of PD. Moreover, PD progression could be favored by oxidative stress, which also contributes to the impairment of the function of the ubiquitin–proteasome system [128,129]. Interestingly, post-mortem dopamine-secreting nerve cells in the pars compacta of the SN (SNpc ba-dc) showed a deficiency of the first mitochondrial complex, resulting from cellular energy metabolism depletion, which is extensively discussed in the next paragraph.

In practice, the diagnosis is based on neurological examination according to International Parkinson and Movement Disorder Society criteria. A good response to dopaminergic therapy supports the diagnosis. Dopaminergic neuroimaging (DaTSCAN) can help to distinguish PD from essential tremor, drug-induced parkinsonism, or conversion disorders, but not from atypical parkinsonisms (such as multi-system atrophy or progressive supranuclear palsy) [130]. In rare cases, PD is genetically determined, with the most commonly known mutations in SNCA, LRRK2, PRKN, PINK1, and GBA genes [131]. In terms of environmental factors’ exposure to toxins, in particular, 1-methyl-4-phenyl-1,2,3,6-tetrahydropyridine (MPTP) and pesticides, as well as repetitive head injuries, can lead to developing PD. On the contrary, smoking, drinking coffee, and taking anti-inflammatory drugs may be protective [132]. Levodopa, dopamine receptors agonists, monoamine oxidase type B inhibitors, catechol-O-methyltransferase inhibitors, and amantadine as dopaminergic therapy are used to treat motor symptoms. In the advanced stage of the disease, to maintain continuous dopaminergic stimulations, three device-aided therapies are available: deep brain stimulation (DBS), levodopa-carbidopa continuous intestinal infusion (Duodopa), and subcutaneous apomorphine infusion (Dacepton) [130].

### 3.1. Disrupted Energy Metabolism in PD-Affected Brain

Observations of PD pathology provide evidence of disrupted mitochondrial dynamics, inhibition of the electron transport chain (ETC), and significantly increased reactive oxygen species [133]. As in AD, glycolysis and glucose uptake are impaired in PD brains, which is observed through reduced cortical glucose consumption. However, unlike in AD, this dysfunction is assumed to be a consequence of mitochondrial dysfunction and it is not a cause as in AD [126]. α-Synuclein is implicated in the progression of mitochondrial dysfunction by binding to the outer mitochondrial membrane and interacting with ATPase [133]. There is evidence for the effect of neurotoxic aggregates on mitochondrial dynamics, particularly mitochondrial fusion, leading to mitochondrial fragmentation [133]. In addition, aggregation of α-synuclein affects the function of PTEN-induced kinase 1 (PINK1), considered a key activator of mitophagy, specifically its stabilization. However, PINK1 stabilization inhibits mitophagy and allows for the maintenance of damaged mitochondria, thereby contributing to ROS overproduction, which leads to intracellular oxidative stress [133]. It might result in mitochondrial DNA abnormalities [134].

Mitochondria isolated from PD patients are deficient in mitochondrial complex I, which plays an important role in dopaminergic neuronal loss [135,136]. It is well known that complex I is a major source of ROS, therefore, its suppression may result in the activation of intrinsic apoptotic pathways. Complex I deficiency in PD patients is noted mainly in the frontal cortex [137]. In addition, elevated levels of oxidized coenzyme Q-10 in the CSF are observed in PD patients, indicating oxidative damage in mitochondria [138]. Oxidative stress and inhibition of complex I are manifested by decreased glutathione levels and inflated iron levels [139]. PD patients also show a deficiency of mitochondrial complex IV (cytochrome c oxidase). Inhibition of complex IV has been implicated in the pathophysiology of the motor disorders observed in PD, with the greatest decrease seen in the frontal cortex and SN [140]. One of the consequences of mitochondrial damage is an impaired calcium metabolism that also contributes to the increase in ROS. However, there is evidence that calcium disruption in the nerve cell is directly influenced by α-synuclein. ASN can form pores in the cell membrane, resulting in an increase in extracellular calcium flow into the cytosol [141]. Increased intracellular calcium levels may lead to increased demand for ATP consumption, which may contribute to the exacerbation of already impaired energy metabolism.

Moreover, similarly to AD, the kynurenine pathway in PD is overexpressed. Disruption of this pathway results in the production of neurotoxic compounds such as 3-HK and QUIN, as well as a reduction in the amount of neuroprotective kynurenic acid. Interestingly, increased levels of kynurenic acid in PD have been associated with the production of cytotoxic metabolites that contribute to neuronal death [142]. As a consequence of mitochondrial dysfunction, a decrease in NAD+ levels was also noted in PD. Research has shown that increased levels of NAD+ can protect against the aggregation of misfolded α-synuclein by improving protein quality control [143,144].

### 3.2. Selected Candidates for Biomarkers Related to Disrupted Energy Metabolism Pathways and Intracellular Homeostasis Imbalance

**Figure 3:**
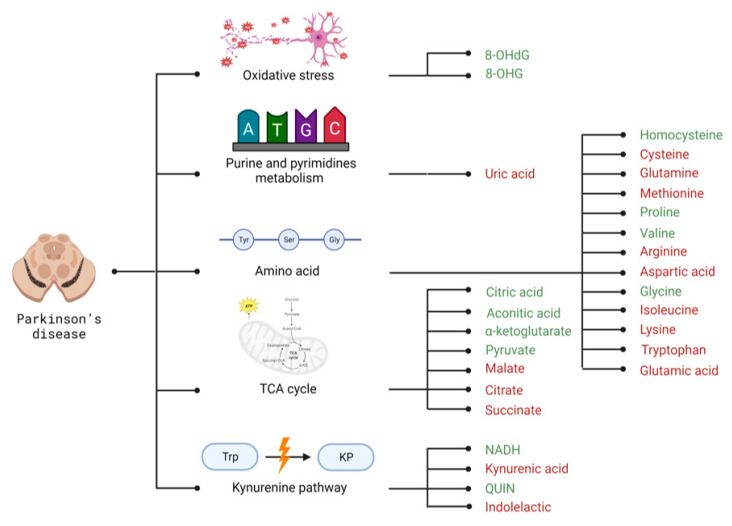
Biomarkers of PD related to energy metabolism disruption (green—increased levels, red—decreased levels).

PD diagnosis is based on clinical examination and there are no specific blood or CSF tests which may help its establishment. Interestingly, in studies conducted on biological material from PD patients 10 amino acids, 2 branched-chain amino acids and 3 Krebs cycle metabolites levels differ significantly from the results obtained for a healthy population [87]. Moreover, similarly to metabolomics of samples collected from AD patients, metabolites indicated that impaired oxidoreductive balance was noted. Selected metabolomics candidates for biomarkers of PD related to disrupted energy metabolism pathways and intracellular homeostasis imbalance are presented in Figure 3.

There is a decrease in plasma arginine levels in PD patients. Arginine is a semi-essential amino acid responsible for normal brain function. In an NADPH-dependent reaction, arginine is oxidized to nitric oxide. Both arginine and nitric oxide are involved in the maintenance of normal synaptic plasticity, neurogenesis, and neuroprotection, and may enhance memory and learning functions [145]. All of the functions described above are impaired in PD, which may suggest a correlation between a decrease in levels of this amino acid and disease progression, however, this has not been confirmed and requires further research to establish the exact role of this amino acid in PD pathology. Furthermore, a decrease in arginine concentration correlates with an increase in serum concentration of another amino acid, glutamate, which is the amino acid that stimulates nitric oxide synthesis [146].

It is well known that the progression of PD is influenced by oxidative stress and redox dysfunction [135]. Another amino acid strongly linked with these processes is glutathione. This amino acid is involved in neuro-oxidative processes, proliferation, and cell differentiation, as well as in the regulation of cell death [147]. PD patients show a significant decrease in glutathione in the SN; however, it has not been established whether the decrease in concentration is a consequence or effect of neuro oxidative stress [87,147]. Furthermore, the decrease in brain glutathione levels is directly linked to the increase in CSF glycine levels, as glycine is one of the three amino acids that build glutathione [87]. Thus, glycine can be easily obtained from PD patients as a more valuable biomarker than glutathione [148].

Acetylation and deacetylation of proteins, particularly histones and non-histone proteins, have also been linked to the pathogenesis of PD [149]. Some studies suggest that the serum level of another amino acid, lysine, is linked to these regulatory processes by influencing the function of the relevant enzymes [150]. However, its deficiency may lead to impaired metabolic processes responsible for the regulation of mood and memory [149]. CSF studies of PD patients support this theory, as levels of this amino acid are significantly lower than in healthy individuals [87,149].

Interestingly, PD studies hypothesized that decreased intestinal transit, length, and motility of the colon may precede the motor symptoms of PD. According to Braak et al.’s hypothesis, the pathological processes in PD start in the intestinal nervous system and/or olfactory bulbs [151]. These disorders are linked with disruption of the gut–microbiota-metabolite axis, as metabolites produced by the bacterium in the gut can promote PD pathologies [152]. It is linked with an increased proline plasma level observed in PD patients, which may indicate the changes in the microbiome that does not use proline as an energy substrate [87,152,153]. Indeed, disturbances in the microbiome are thought to promote an inflammatory process, leading to the formation of amyloid-β and Lewy body deposits in Meissner’s and Auerbach’s plexuses, and further, this pathology is believed to penetrate the brain via a prion-like mechanism along pathway X [154]. In addition, other studies highlighted the decreased aspartate and increased aspartate levels in CSF of PD patients; however, no direct associations of changes in the concentration of these amino acids with PD progression have been discovered [87].

As in AD, pathways linked with tryptophan metabolism are also impaired in PD. A decrease in tryptophan levels is observed in the CSF of PD patients [87]. This is attributed to overexpression of the pathway and increased consumption of the substrate, which is transformed into toxic metabolites contributing to the progression of PD pathology [87,142]. Lower levels of kynurenic acid and kynurenic acid/kynurenine ratio and higher levels of QUIN and QUIN/ kynurenic acid ratio were reported in the plasma of PD, also indicating a biased kynurenine pathway toward producing oxidative stress and excitotoxicity [155,156]. However, there are contrary data associated with tryptophan metabolism with aging [157,158]. Apart from kynurenine metabolites, Shao Y et al. also observed a significantly decreased level of indoleacetic acid, another tryptophan catabolite, in PD [159]. Activation of the kynurenine pathway was also confirmed by increased plasma NADH levels in PD patients relative to healthy control. Interestingly, it was associated with increased nicotinamide phosphoribosyl transferase (that converted nicotinamide to nicotinamide mononucleotide) in PD plasma [160]. Nevertheless, levels of nicotinamide metabolites have not been studied yet in PD.

Additionally, homocysteine plasma levels in PD patients are higher compared to plasma levels of this compound in healthy individuals [32,87]. It has been suggested that homocysteine drives the progression and development of PD. Moreover, PD plasma homocysteine levels are also strongly correlated with vitamins B_12_ and B_9_, which are cofactors for proper homocysteine metabolism [161]. These results show a possible therapeutic approach [162,163]. High levels of homocysteine interfere with the apoptosis process and induce oxidative stress [164]. Although homocysteine was also excessively produced during the levodopa treatment in PD patients, this highly decreases its usefulness as a PD biomarker [163].

Glutamic acid is the last of the selected amino acids that are disrupted in PD. The level of this amino acid correlates with neuronal migration and survival [165]. A decrease in the plasma levels of this amino acid is observed in PD patients, which may be linked with increased neuronal apoptosis in PD [165]. Moreover, it is in line with diminished TCA cycle metabolites (citrate, malate, and succinate) levels in PD patients that confirm the disruption of this cycle, mitochondrial damage, and lack of ATP production, which may promote disease progression [166,167,168]. Recently, the LC–MS metabolomics-based study has shown altered plasma levels of aconitic acid in patients with PD [168,169]. This is in line with the results was obtained by Wu et al., who observed elevated levels of citric acid in the CSF of patients with PD [148]. In addition, elevated levels of other compounds which are part of the TCA cycle, α-ketoglutarate and pyruvate, were observed by Willkommen et al. in the CSF of PD patients [167]. 

Similarly to AD, markers of PD progression may become markers of oxidative stress, which is increased in PD but is not a specific sign of the disease [170]. Another compound that shows biomarker potential is uric acid (also highlighted in AD sections). Its concentration in the SN in PD patients is reduced. However, in this study, inverse correlation studies have found an increase in serum in 8-OHdG, nitrotyrosine, or oxygen-free radicals [170], and PD progression of serum uric acid with disease duration has been shown. It suggests that although reduced uric acid levels may precede the appearance of symptoms of PD, the progression of the disease might be associated with a further decrease. Indeed, studies indicate that higher uric acid concentrations in patients slow the progression of PD [171,172].

Nevertheless, although the mentioned metabolites are encouraging, there are some challenges to overcome. Rebai et al. suggested that PD patients’ metabolomic profile might depend on glutathione S-transferase polymorphism [173]. Specifically, the glutathione S-transferase theta 1 positive group of patients exhibited increased plasmatic levels of several organic acids (e.g., citric acid) while exhibiting decreasing levels of proline and valine [173]. It has been also reported that PD patients showed differences between males and females in epidemiological and clinical characteristics, sensitivity to risk factors, response to treatments, and a concentration of metabolites, including amino acids [174]. Moreover, the concentration of amino acids may also be dependent on the composition of the microbiota of patients with PD, which affects amino acids biosynthesis, absorption, and transformation in the gastrointestinal tract [175]. Thus, similarly to AD, this group of metabolites might not be a suitable biomarker for PD.

## 4. Huntington’s Disease

Huntington’s disease (HD) is an autosomal dominant hereditary neurodegenerative disorder. The clinical spectrum includes choreatic movements (but also dystonia, tics, or parkinsonism), depression with a high rate of risk of suicide, and dementia [176,177,178]. Unlike AD and PD, HD is not a disease of old age, as the first symptoms occur between the ages of 30 and 50, and the disease can affect a 2-year-old as well as an 80-year-old. HD is estimated to affect around half a million people worldwide [179]. 

HD results from a mutation in the IT15 gene, which is located on the short arm of chromosome 4, leading to multiple trinucleotide repeats of the N-terminal region of the huntingtin (Htt) protein [180]. The mutant huntingtin protein (mHtt), in which the glutamine I polyglutamine (poly Q) residue has been elongated, forms insoluble and cytotoxic aggregates in the nucleus and cytoplasm, in which mHtt mainly adopts a beta-card structure. In addition, mHtt interacts with other proteins that are involved in transcription, the cell cycle, or cell metabolism, thereby disrupting the homeostasis of these processes and ultimately leading to cell death [181]. The ubiquitin–proteasome system (UPS), which is responsible for the autophagy process of misfolded proteins, is also disrupted in HD. Therefore, mHtt cannot be degraded [182,183]. In addition, mHtt, by interacting with autophagy receptors, blocks the possibility of their binding to damaged mitochondria [184]. Moreover, high levels of BNip3, which is a pro-apoptotic protein of the BH3 group belonging to the Bcl-2 family, were detected in HD patients. These proteins induce the formation of pores in the mitochondrial membrane through which cytochrome c is released into the cytoplasm, which then activates the caspase cascade [185]. mHtt also affects the balance in cell organelles, especially mitochondria that are responsible for maintaining cellular energy metabolism.

Clinical diagnosis is based on genetic testing. CAG repeat extension ≥ 36 is pathogenic, with full penetrance ≥ 39 repeats [186]. Positive family history may not always be present due to, for example, non-paternity, parents dying young, or de novo cases [187]. Disease-modifying therapy is not available. Tetrabenazine and Deutetrabenazine are licensed to treat chorea [188]. In clinical practice, neuroleptics are also used for this indication. They may also be helpful in treatment of aggressive behavior or psychosis. The treatment of depression and anxiety includes selective serotonin reuptake inhibitor (SSRIs) and psychotherapy [182,186].

### 4.1. Disrupted Energy Metabolism in HD-Affected Brain

Fluorodeoxyglucose positron emission tomography provides evidence of impaired energy metabolism in the cerebral cortex and caudate in pre-symptomatic HD. Furthermore, studies of mitochondrial oxidative metabolism in the striatum of pre-symptomatic HD patients highlighted selective defects in striatal glycolysis in the early stages of HD, suggesting that metabolic deficits are present in the early disorder of HD and occur before the onset of clinical symptoms. In addition, studies using magnetic resonance spectroscopy (MRS) imaging provide evidence of impaired glycolysis and TCA cycle function. It is hypothesized that inhibition of mitochondrial complex II, impaired calcium metabolism, increased levels of oxidative stress, and dysregulation of factors controlling mitochondrial biogenesis may contribute to HD pathology [189].

As a result of increased carbonylation of mitochondrial enzymes, their activity is reduced, leading to a decrease in energy production in the striatum. Evidence for the inhibition of mitochondrial complex II by mHtt is provided by post-mortem studies of the striatum of HD patients. It is known that, in the striatum, the mHtt lead to an increased sensitivity to oxygen deprivation of striatal cells by changing mitochondrial membrane potential. The mHtt protein also disrupts the cellular pathways that regulate calcium levels. Disruption of calcium metabolism can unload the mitochondrial membrane potential, releasing cytochrome c, ultimately leading to neuronal cell apoptosis [190]. As for the earlier-discussed neurodegenerative diseases, the disturbance of energetic metabolism in HD might also be caused by oxidative stress, which was indicated by increased levels of 8-hydroxydeoxyguanosine (8-OHdG) in the striatum. Oxidative stress may contribute to increased mutant huntingtin aggregation [191].

Another process that is disrupted during HD progression is mitochondrial fission. Indeed, increased levels of mitochondrial fission regulator, dynamin-related protein 1 (Drp1), and mitochondrial fission 1 protein (Fis1) have been observed in HD [192]. There is also an alteration in the activity of AMPK, which is one of the main energy sensors. In the early stages of the disease, increased AMPK activity is observed, which decreases as the disease progresses. However, there is no conclusive evidence for a negative effect of AMPK, as in the early stages of the disease its activity induces defense responses in response to mHtt-induced energy deficits, whereas, in advanced stages of the disease, it may contribute to cell apoptosis [193]. Another metabolic pathway that is impaired in the HD is kynurenine metabolism. mHtt sensitizes N-methyl-D-aspartic acid (NMDA) receptors to neurotoxic metabolites: 3-HK and QUIN. Furthermore, disruption of its pathway promotes glutamate-induced excitotoxicity [194]. 

### 4.2. Selected Candidates for Biomarkers Related to Disrupted Energy Metabolism Pathways and Intracellular Homeostasis Imbalance

**Figure 4:**
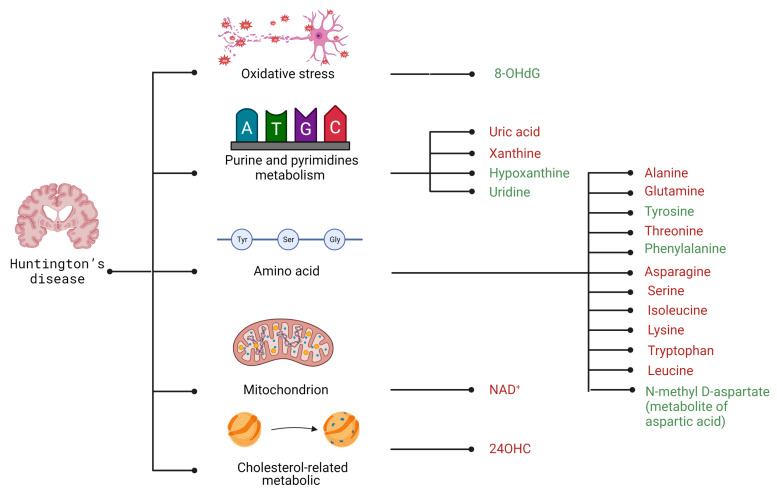
Biomarkers of HD related to energy metabolism disruption (green—increased levels, red—decreased levels).

Unlike the neurodegenerative diseases listed above, there is an excellent genetic parameter to confirm disease onset in HD (the CAG repeat size within the HTT gene); however, it is necessary to find biomarkers that reliably define HD status or determine and predict the phenoconversion of the disease and the rate of progression [195]. The proposed metabolomics biomarkers related to disrupted energy metabolism in HD are depicted in Figure 4.

Post-mortem brain examinations showed significant changes in the levels of methionine, lysine, leucine, threonine, and isoleucine. Their levels were markedly reduced, particularly in the hippocampus [196]. It is well known that these amino acids might be used as substrates in the TCA cycle (via acetyl-CoA and succinyl-CoA) [196]. Thus, reduced levels of these compounds may indicate an attempt to supply the substrates for the TCA cycle to maintain proper cellular energy levels. Other (determined CSF from HD patients) amino acids whose levels differed markedly from the control group were asparagine and serine. Both showed lower levels [197]. Asparagine is a metabolic product of aspartic acid, which, as a result of the formation of oxaloacetate, may reflect a disruption of the TCA cycle. Thus, one may conclude that easily quantifiable asparagine may become a biomarker informing TCA disruption [198]. Interestingly, another aspartic acid derivative, N-acetyl aspartate (NAA) serum levels increase, emerged in HD patients. It was positively correlated with the global motor impairment score and functional decline. Nevertheless, the interpretation of this finding is presently limited by the scarce knowledge about the basal mechanism of serum NAA production, and the very small cohort of patients, evaluated at different ages and stages of HD, and in some cases during neuroleptic treatment [199].

In 2022, the multi-omics of human, mice, and yeast HD model systems also underlined the deregulation of glycolysis, TCA cycle, and pyruvate metabolism in HD datasets, which was in line with previous studies [200,201,202]. This study also indicated lower levels of alanine in HD patients, as well as in HD mouse model systems. Moreover, it highlighted contradictory data for glutamine levels, which are elevated in mouse and yeast metabolomics datasets but decreased in those from HD patients. However, in experimental studies, the addition of glutamine in the yeast model of HD led to the significantly increased aggregation of HTT protein. Previous HD biomarker studies have shown decreased levels of alanine and isoleucine in HD patients, whereas alanine and leucine levels are elevated in pre-symptomatic cases [203,204]. It is well known that deregulation of alanine, aspartate, and glutamate metabolisms is attributed to decreased glucose levels, which is concomitant with the energy deficits in the brain and peripheral organs of HD patients [87]. Taken together, alanine might be an important metabolite to modulate HTT protein aggregation in HD and, therefore, HD progression markers. Moreover, besides alanine, another amino acid, serine levels might reflect neuronal cell degradation. The concentration of this amino acid participates directly or indirectly in the changes associated with HD pathogenesis, meaning that its fluctuating level may help to monitor disease progression [197,205]. 

Moreover, there is a definite increase in tryptophan, phenylalanine, and tyrosine levels in the brain of HD subjects compared to levels of these amino acids in controls [196]. It can be concluded that tyrosine may be a specific biomarker of HD because it has an affinity to binding to the wild-type HTT protein to thyroid hormone receptor-α1 [206,207,208,209]. It is well known that tryptophan accumulation is associated with the disruption of the kynurenine pathway. It can be assumed that indoleamine 2,3-dioxygenase (IDO), which mediates the conversion of tryptophan to kynurenine, is impaired in this brain area [210]. Additionally, in HD patients’ blood, an increased ratio of kynurenine to tryptophan and a significant decrease in tryptophan was observed [211]. However, the change in this ratio is observed in symptomatic patients, indicating that the kynurenine pathway disorder occurs in the later stages of the disease and may be a consequence rather than a cause [212]. 

In 2020, McGarry et al., contrary to previous studies suggesting decreased levels of NAD+ would be expected with progressive neurodegeneration, highlighted higher circulating CSF levels of NAD+ correlated to worsening clinical status [213]. The reason for this is not clear. Considerations included decreased levels or function of CD38, a highly expressed glycoprotein in neurons and astrocytes that generates cyclic ADP-Ribose (cADPR) from NAD+ molecules, or a proportional decrease in the intracellular concentration of NAD+ [214]. The latter case would be consistent with previous reports that NAD+ is preferentially released from intracellular stores in conditions of cell stress or inflammation [215]. On the other hand, our previous study indicated similar NAD+ blood levels in HD symptomatic patients and healthy controls [216].

In HD, as in other earlier-mentioned neurodegenerative diseases, increased oxidative stress is observed [211]. While uric acid acts as a neuroprotector, reduced uric acid levels are observed in the plasma of HD patients compared to healthy individuals. These results were also confirmed by post-mortem analyses of HD brains [217]. Furthermore, uric acid levels decrease with disease progression [218]. It was also measured in plasma and saliva using a colorimetric enzymatic reaction kit [217]. Since it can be determined from readily available material, it has the predisposition to be a good indicator of HD. 

Another biomarker of oxidative stress whose level was elevated in HD patients’ blood is 8-hydroxy-2-deoxyguanosine (8-OHdG) [211]. However, 8-OHdG still requires more research, as the results obtained so far are inconclusive [219]. Another compound whose levels in HD are significantly out of line with the results obtained for the control group is xanthine, whose plasma levels in HD are decreased [218]. Furthermore, our previous study indicated higher levels of other compounds related to purine and pyrimidine metabolism, hypoxanthine, and uridine in HD patients relative to healthy controls [216,220]. 

Additionally, studies have implicated disruptions in cholesterol-mediated metabolic pathways in the brain as a contributing factor to neurodegenerative disease pathogenesis. In light of this, research is investigating the utility of 24(S) hydroxy cholesterol (24OHC), a key cholesterol metabolite, as a diagnostic biomarker for these diseases. Noteworthily, a correlation has been observed between decreased plasma levels of 24OHC in HD patients and reduced volume of the caudate nucleus [211]. It is hypothesized that the level of this metabolite is correlated with the level of nerve cell loss [221]. Therefore, it may qualify as a biomarker of disease progression. However, validation of this biomarker requires long-term follow-up of patients to determine changes in 24OHC levels during disease evaluation [211,221].

It is widely acknowledged that changes in energy brain metabolic processes resulted in changes in peripheral levels of metabolites in HD patients. Still, additional multi-omics and multi-model studies are needed to identify a metabolite that can be used to predict the onset of HD and its progression rate. Thus, despite the presence of promising metabolites related to HD onset and the advancements in developing methods for metabolomics analysis, it remains challenging to establish reliable biomarkers for the clinical management of rare diseases such as HD.

## 5. Discussion

Metabolomics is a field of the “omics” sciences that examines global changes in metabolites and metabolic networks. It has the potential to identify, at a systems level, the molecular signatures, interactions, and metabolic pathways that are involved in complex diseases, such as neurodegenerative disorders, which have multi-factorial causes. It has been applied in both preclinical and clinical research. It helps characterize and distinguish the pathological signatures and potential biomarkers of Alzheimer’s Disease, Parkinson’s disease, and Huntington’s disease. There is a continuously expanding array of fluid biomarkers that can be useful in ND research, which has the potential to fill crucial gaps in both research and clinical practice. However, as we presented in this work, currently only a limited number of target molecules linked with impaired energy metabolism have been associated with the pathogenesis of these neurodegenerative diseases.

**Figure 5:**
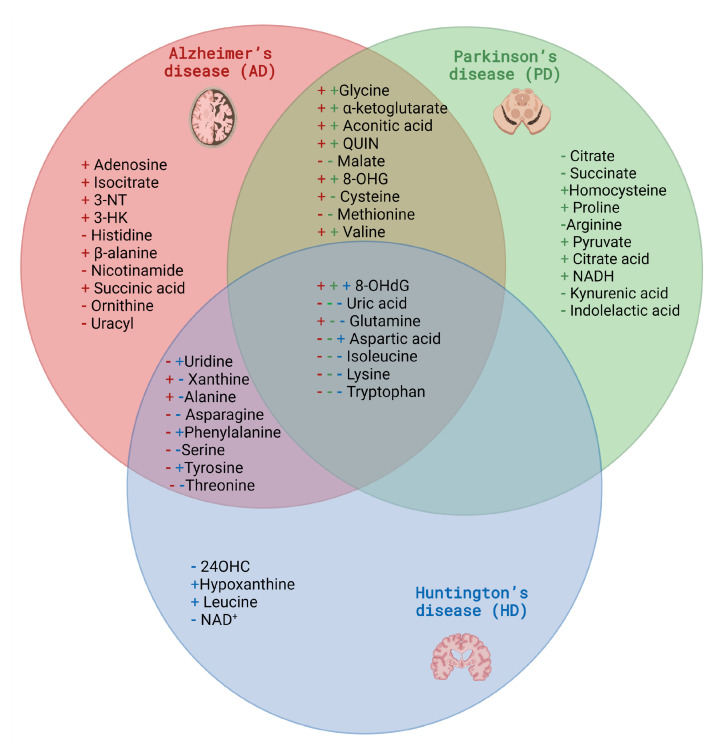
Comparison of disturbances in concentrations of metabolites associated with impaired energy metabolism in AD, PD, and HD.

Disrupted brain oxidative metabolism, and thus energy metabolism depletion (caused by impaired TCA cycle or deterioration in mitochondrial function or oxidative stress) in neurodegenerative diseases, result to changes in serum, plasma, blood, or cerebrospinal fluid levels of oxidative stress markers, TCA cycle, purine catabolism, and kynurenine pathway metabolites. Furthermore, due to the essential role of amino acids in the central nervous system (neurotransmitters, regulators of metabolism, and neuromodulators), the neurodegenerative diseases investigated in this work were also characterized by impaired amino acid metabolism that affects its peripheral concentration. Some similarities and differences between the disease metabolomics footprint signatures are presented in Figure 5 and Table 1.

The shared metabolites linked with disrupted energy metabolism in all investigated neurodegenerative diseases—AD, PD, and HD—are 8-OHdG (increased), uric acid, lysine isoleucine, and tryptophan (decreased). One may conclude that this group of metabolites could be useful in ND diagnosis. Nevertheless, its levels were also disrupted in other non-CNS diseases. Increased 8-OHdG levels were noted in cardiovascular diseases, kidney disease in individuals with type 1 diabetes, and colorectal or serous ovarian cancer [222,223,224,225]. Hypouricemia may be caused by decreased uric acid production, uric acid oxidation due to treatment with uricase, or decreased renal tubular reabsorption due to inherited or acquired disorders [226,227]. Serum lysine levels (besides other AA) seem to be stable during diet changes, for example, the oxidation of lysine is at similar levels in humans with protein restriction [228]. Nevertheless, there are data suggesting that the synthesis of lysine by intestinal microflora with absorption by the host has also been a potentially important source of lysine [229]. Thus, its serum level may also depend on the functionality of the patient’s intestine. Lysine levels were also decreased in patients with hematological disease—acute myeloid leukemia [230].

As mentioned earlier, lower isoleucine levels were strongly associated with an increased risk of AD in a combined meta-analysis with a replication sample. In HD studies, diminished isoleucine levels were indicated in HD-affected human brains, as well as in the HD patient’s serum compared to healthy controls [43]. In addition, the levels of isoleucine negatively correlated with UHDRS values and the number of CAG repeats [43]. Interestingly, a recent study found that plasma isoleucine level was decreased in PD patients and correlated with clinical characteristics. Compared with early PD patients, isoleucine levels were even lower, and microbial composition was altered in advanced PD patients. The mentioned study also suggested that the disturbances of plasma isoleucine and other BCAAs in PD patients may be related to the gut microbiota and exacerbated with PD severity [231]. Nevertheless, lower serum isoleucine levels were also associated with depression, multiple sclerosis, and peripheral tissue disorders such as uterine leiomyomas [232,233,234], which reduces its usefulness as a NDs indicator. 

In three of the investigated NDs, lower tryptophan levels were noted. Tryptophan and its oxidative metabolic pathway KP was a promising object of interest, mainly in the HD biomarkers study, although Rodrigues et al. recently reported no significant change in most KP metabolites from CSF or plasma among manifest, premanifest HD patients, and healthy controls, and no associations with any clinical or imaging signs. They also suggested that the change of peripheral KP metabolites is supposed to be interpreted cautiously because central KP metabolites have different abilities to cross BBB, and this raises the question of whether blood is an appropriate medium to reflect the alterations of those complex components in KP related to neuropathology in HD or other NDs [235].

In contrast, glutamine levels increase in AD and decrease in HD and PD. Nevertheless, it should be highlighted that glutamine level examination in AD patients presents many controversial findings [43,87,230,236]. One of the possible reasons for these inconsistencies could be that the criteria for diagnosis of probable AD in early studies included only clinical features, which could lead to greater heterogeneity amongst individuals included in the studies and, hence, greater variability in results from CSF analysis. Nevertheless, one above-mentioned study which indicated increased CSF glutamine levels examined patients with probable AD only positive for the biomarker amyloid tau index (IATI) (thus showing clear evidence of both amyloid-β and tau neuropathology). Interestingly, in this study, glutamine levels were inversely correlated with individual IATI values across investigated groups of subjects [236]. According to incoherent CSF data and no data from AD patient’s serum, more large population studies in AD are needed to elucidate the role of glutamine as a biomarker of AD.

Common metabolites for AD and HD disease are uridine, xanthine, alanine, asparagine, phenylalanine, serine, tyrosine, and threonine. In both AD and HD patients, a decrease in levels of asparagine, serine, and threonine is observed, while in AD a decrease in levels of xanthine and alanine is observed in HD. In the levels of the others, an inverse relationship is observed with an increase in levels of HD and a decrease in AD. Interestingly, the most similar metabolomics footprint is observed in AD and PD (the exception is cysteine, which increases AD and decreases PD), while no related metabolites are observed between PD and HD. Although AD and PD have markedly different clinical and pathological features, many mechanisms involved in AD and PD may be the same, such as common genetic determinants, presence of α-synuclein protein and tau protein, oxidative stress induction, mitochondrial dysfunction, iron, or, as presented in this review, energy metabolism derangements [237,238]. The highest number of compounds allowing for disease differentiation is observed in AD, while the lowest is in HD. It suggested that more metabolomics studies in HD patients are needed to fully understand and successfully compare HD metabolomics profiles with other NDs.

**Table 1:** Comparison of disturbances in concentrations of metabolites associated with impaired energy metabolism in AD, PD, and HD (+ increased level, - decrease level, x—no information).

Group of Metabolites	Compound	AD	PD	HD
Amino acids	Alanine	+ [87]	x	- [204]
	Arginine	x	- [146]	x
	Asparagine	- [88]	x	- [197]
	Aspartic acid	- [88]	- [87]	+ [194,199]
	Cysteine	+ [87]	- [87]	x
	Glutamic acid	x	- [87]	x
	Glutamine	+ [87]	- [87]	- [203]
	Glycine	+	+ [87]	x
	Histidine	- [87]	x	x
	Homocysteine	x	+ [164]	x
	Isoleucine	- [99]	+ [87]	- [43,67]
	Leucine	x	x	+ [196]
	Lysine	- [88]	- [87]	- [196]
	Methionine	- [87]	- [87]	x
	Ornithine	- [88]	x	x
	Phenylalanine	- [88]	x	+ [196]
	Proline	x	+ [87]	x
	Serine	- [88]	x	- [197]
	Threonine	- [88]	x	- [196]
	Tryptophan	- [88]	- [87]	+ [211]
	Tyrosine	- [88]	x	+ [196]
	Valine	+ [99]	+ [87]	x
	β-alanine	+ [87]	x	x
Cholesterol-related metabolic	24OHC	x	x	- [211]
Kynurenine pathway	3-HK	+ [102]	x	x
	Indolelactic	x	- [160]	x
	Kynurenic acid	x	- [155]	x
	QUIN	+ [102]	+ [155]	x
Mitochondrion	NAD^+^	x	x	- [214]
	NADH	x	+ [158]	x
Oxidative stress	8-OHdG	+ [106]	+ [169]	+ [211]
	8-OHG	+ [106]	+ [169]	x
	3-NT	+ [105]	x	x
Purine and pyrimidines metabolism	Adenosine	+ [87]	x	x
	Hypoxanthine	x	x	+ [216]
	Uracil	- [88]	x	x
	Uric acid	- [87]	- [87]	- [218]
	Uridine	- [87]	x	+ [216]
	Xanthine	+ [87]	x	- [218]
TCA cycle	Aconitic acid	+ [101]	+ [167]	x
	Citrate	x	- [87]	x
	Citric acid	x	+ [148]	x
	Isocitrate	+ [87]	x	x
	Malate	- [89]	- [87]	x
	Pyruvate	x	+ [87]	x
	Succinate	x	- [87]	x
	Succinic acid	+ [101]	x	x
	α-ketoglutarate	+ [87]	+ [167]	x
Other	Nicotinamide	- [100]	x	x

It has also been mentioned that metabolomics profiling of NDs is associated with many methodological challenges. The abundance of some markers in humans is exceedingly varied, as NDs are exceedingly complex and heterogeneous, and some of these differences may be biological. Moreover, this group of diseases often also appears with other peripheral tissue metabolic deteriorations that are caused independently (co-morbidities that are frequently present in the elderly population) or accompanied by diseases such as HD-related cardiomyopathy and myopathy. Thus, the peripheral concentration of metabolites will be the result of the disorders noted not only in the central nervous system but also in other non-neuronal tissues, e.g., skeletal muscle or heart. One of the solutions to overthrow this problem might be a measurement of metabolites only in CSF, but the invasiveness of its collection is not without limitations. On the other hand, blood sampling can easily be incorporated into regular clinical visits with minimal time investment. However, potential confounding factors such as variations in sample collection and storage, different methodologies, and small sample populations require standardized procedures for this type of sample collection, processing, and analysis. The diversity of study specimens and used metabolomics methods can contribute to the versatility or, as presented in this review, sometimes some contradictions in ND metabolomics biomarker studies. Thus, it is important to determine the disturbances in investigated metabolites or related pathways in different tissue types or materials collected from NDs patients. The correlation of the selected metabolite or pathway with the disease onset or progression must also be confirmed with another analytical method.

Despite advances in our understanding of the relationships between metabolomics data and physiological processes in neurodegenerative diseases, much remains unknown about the underlying biochemical mechanisms. In recent years, many studies have converged on the idea that the mechanism behind neurodegenerative diseases involves the rupturing of intracellular vesicles and incorrect protein aggregation [239]. Nevertheless, in the case of some metabolites, it is still not clear whether its disrupted level is a cause or an effect of the NDs. Metabolomics, connected with basic science research, aims to shed light on the role of metabolites in biological processes and to build a more comprehensive understanding of these processes. However, so far, the interpretation of biological processes remains a challenge in metabolomics research.

## 6. Conclusions

In conclusion, due to the increasing interest of researchers in improving the diagnosis and predicting the onset of NDs, numerous studies have been conducted on metabolomics biomarkers for these diseases. However, none of selected metabolomics biomarker have been specific for only one particular ND and were not related with other non-CNS diseases. Thus, in the future, comprehensive analysis of chiral metabolite isoforms, as well as their oxidized or chemically modified forms, may become increasingly important in the diagnosis and prediction of NDs.

Due to the multifaceted nature of NDs and the different sensitivities of each biomarker, it is unlikely that a single metabolomic biomarker will be identified. Therefore, in our opinion, the most valuable biomarkers for NDs studies will be the metabolite or groups of metabolites combined with other neuroimaging or molecular techniques. This emphasizes the importance of collaborative research and standardization of protocols throughout the field of ND’s biomarker research.

Based on data collected from preclinical and clinical NDs studies, changes in the energy metabolism and the metabolomic footprint may be different in various disease stages. This could be an advantage (it might help monitor the disease progression), but only with proper clinical neuroimaging, psychological tests, and data collection in large research groups, collected not only from NDs patients but also from a healthy population. Metabolite concentrations might be affected by diet, drugs, environment, and circadian rhythms. Thus, similarly to clinical data, information about taken pharmaceuticals, supplements, or special diets should be routinely collected during an investigation of the possible metabolomic biomarkers of NDs.

It has to be also mentioned that, unfortunately, all current metabolomics studies have relatively small sample sizes, which will limit our ability to further correlate and replicate. Additionally, only some of them investigated the concentration of selected metabolite within the disease and symptoms progression, which is crucial for ND’s prediction and prognosis. To overcome this, more follow-up studies in larger cohorts and larger data are needed.

Thus, besides this work indicating that biochemical parameters of brain energy metabolism disruption (obtained with metabolomics) may have a potential application as a diagnostic tool for diagnosis, prediction, and monitoring of the effectiveness of therapies for NDs, more studies are needed to determine the sensitivity of the indicated biomarkers or group of metabolites proposed as candidates. Presumably, to attain clinically applicable results, the integration of metabolomics with other “omic” techniques might be required.

## Abbreviations

The following abbreviations are used in this manuscript:

24OHC	24(S) hydroxy cholesterol
3-HK	3-hydroxyquinurenine
3-NT	3-Nitrotyrosine
8-OHdG	8-hydroxy-2′-deoxyguanosine
8-OHG	8-hydroxyguanosine
AcAc	acetoacetate
AcAc-Co-A	acetoacetyl-CoA
ACAT	acyl-CoA: cholesterol acetyltransferase
AD	Alzheimer’s disease
ADNI	Alzheimer’s Disease Neuroimaging Initiative
ALT	alanine aminotransferase
AMPK	adenosine monophosphate-activated protein kinase
ApoE	apolipoprotein E
APP	amyloid precursor protein
ASN	alpha-synuclein proteins
ATP	adenosine triphosphate
Aβ	beta-amyloid peptide
AβPP	beta-amyloid precurso peptide
BBB	blood–brain barier
BCAA	branch-chained amino acid
BHB	beta-hydroxybutyrate
BHD	betahydroxybutyrate dehydrogenase
cADPR	cyclic ADP-Ribose
cBCAT	cytosolic branched-chain aminotransferase
CNS	central nervous system
COX	cytochrome oxidase
CSF	cerebro-spinal fluid
DBS	deep brain stimulationz
Drp1	dynamin-related protein 1
ETC	electron transport chain
FDG-PET	fluorodeoxyglucose-PET
FFA	fatty acid
Fis1	mitochondrial fission 1 protein
fructose-2,6-P2	fructose-2,6-bisphosphate
G3PDH	glyceraldehyde-3-phosphate dehydrogenase
GABARAPL1	γ-aminobutyric acid receptor-associated protein-like 1
GDH	glutamine dehydrogenase
GLUT1	neuronal glucose transporter 1
GLUT3	neuronal glucose transporter 3
GP	glycogen phosphorylase
GS	glutamine synthase
GS	glycogen synthase
GSK-3	GS kinase-3
HD	Huntington’s disease
Htt	huntingtin protein
IDH	isocitrate dehydrogenase
IDO	indoleamine 2,3-dioxygenase
IIS	inhibition of the insulin
IR	insulin receptor
KB	ketone bodIES
KP	kynurenine pathway
LB	Lewy bodies
LDH	lactate dehydrogenase
LNAA	large neutral amino acid
MAP	microtubule-associated protein
mBCAT	mitochondrial branched-chain aminotransferase
MCT	monocarboxylate transporter
MHC	tissue compatibility system
mHtt	mutant huntingtin protein
MPTP	1-metylo-4-fenylo-1,2,3,6-tetrahydropirydyny
MRI	magnetic resonance imaging
MRS	magnetic resonance spectroscopy
NAA	N-acetyl aspartate
NAD+	nicotinamide adenine dinucleotide
NDs	neurodegenerative diseases
NFT	neurofibrillary tangle
NMDA	N-methyl-D-aspartic acid
PD	Parkinson’s disease
PDH	pyruvate dehydrogenase
PDHC	pyruvate dehydrogenase complex
PFK	phosphofructokinase-1
Pfkfb3	6-phosphofructose-2-kinase/fructose-2,6-bisphosphatase-3
PiB-PET	Amyloid Pittsburgh compound B-PET
PINK1	PTEN-induced kinase 1
poly Q	glutamine I polyglutamine
PP-1	protein phosphatase-1
PPP	pentose–phosphate pathway
PSEN-1	presenilin-1
PSEN-2	presenilin-2
PTG	protein targeting to glycogen
QUIN	quinolinic acid
ROS	reactive oxygen species
SCOT	succinyl-CoA:3-ketoacid coenzyme A transferase
SN	substantia nigra
SNpc	substantia nigra pars compacta
SSRI	includes selective serotonin reuptake inhibitor
STBD1	starch-binding domain-containing protein 1
TCA	tricarboxylic acid cycle
TNF-α	tumor necrosis factor-alpha
UA	uric acid
UPS	ubiquitin–proteasome system
α-KG	α-ketoglutarate
α-KGDH	α-ketoglutarate dehydrogenase
α-KIC	α-ketoisocaproate
